# Role of Silica Nanoparticles in Abiotic and Biotic Stress Tolerance in Plants: A Review

**DOI:** 10.3390/ijms23041947

**Published:** 2022-02-09

**Authors:** Lei Wang, Chuanchuan Ning, Taowen Pan, Kunzheng Cai

**Affiliations:** 1 Guangdong Provincial Key Laboratory of Eco-Circular Agriculture, Guangzhou 510642, China; kishi218@163.com (L.W.); ningcc@scau.edu.cn (C.N.); pantaowen123@126.com (T.P.); 2 Key Laboratory of Tropical Agricultural Environment in South China, Ministry of Agriculture, Guangzhou 510642, China; 3 College of Natural Resources and Environment, South China Agricultural University, Guangzhou 510642, China

**Keywords:** silica nanoparticles, uptake, growth promotion, disease resistance, salinity, drought, heavy metal stress

## Abstract

The demand for agricultural crops continues to escalate with the rapid growth of the population. However, extreme climates, pests and diseases, and environmental pollution pose a huge threat to agricultural food production. Silica nanoparticles (SNPs) are beneficial for plant growth and production and can be used as nanopesticides, nanoherbicides, and nanofertilizers in agriculture. This article provides a review of the absorption and transportation of SNPs in plants, as well as their role and mechanisms in promoting plant growth and enhancing plant resistance against biotic and abiotic stresses. In general, SNPs induce plant resistance against stress factors by strengthening the physical barrier, improving plant photosynthesis, activating defensive enzyme activity, increasing anti-stress compounds, and activating the expression of defense-related genes. The effect of SNPs on plants stress is related to the physical and chemical properties (e.g., particle size and surface charge) of SNPs, soil, and stress type. Future research needs to focus on the “SNPs–plant–soil–microorganism” system by using omics and the in-depth study of the molecular mechanisms of SNPs-mediated plant resistance.

## 1. Introduction

The Food and Agriculture Organization (FAO) estimates that in 2050, the global population will increase to 9 billion, and global food production should increase by 70% to meet the growing population’s demand for food [1,2]. At present, biotic and abiotic stressors such as drought, extreme climate, salt, diseases, and pests cause crop loss worldwide. The influence of abiotic stresses on crop production causes an annual loss of crop yield of 51–82% [3]. To increase crop yield, farmers frequently use pesticides and fertilizers, creating a huge threat to the ecological environment. Therefore, environmentally friendly technologies should be developed to help plants overcome biotic and abiotic stresses and ensure optimum crop yield and agricultural sustainability.

Nanotechnology application in agriculture is an emerging interdisciplinary field, which plays a positive role in promoting plant growth and conferring stresses tolerance in plants and has a great potential for applications in agriculture [4,5,6]. Nanoparticles (NPs) are small particles with a size range of 1–100 nm. In comparison with bulk compounds, NPs exhibit different physical and chemical properties in terms of their surface, optical, thermal, and electrical properties [7]. NPs have unique physiological characteristics, large surface-area-to-weight ratios, and small sizes, which can increase their solubility and transportation speeds within plants [8]. Several nanomaterials such as Fe_3_O_4_, MgO, SiO_2_, and CeO_2_ are beneficial for plant growth, playing an important role in promoting seed germination, increasing plant resistance, degrading pesticide residues, and enhancing soil quality [9,10,11,12].

The hydrolyzed product of silica nanoparticles (SNPs) is monosilicic acid (H_4_SiO_4_) [13]. Silicon is the second most abundant element in the earth’s crust and plays a pivotal role in many biogeochemical processes. Silicon strengthens the physical barrier of plants through its deposition onto plant cell walls, thus promoting the ability of lodging tolerance and disease resistance [14]. The applications of silicon and SNPs reduce the oxidative stress response by priming defense reactions under biotic and abiotic stresses [15,16,17,18]. Moreover, SNPs have a small particle size and easily penetrate the plant cell wall and the organelles [19]. In comparison with bulk materials, NPs have a high surface-area-to-volume ratio, thus improving their reactivity and biochemical activity [20,21]. The SNPs’ accumulation in corn is 9.14% higher than for bulk silica [22]. Therefore, SNPs may be more effective than bulk silica in alleviating different adversity stresses [18,23,24,25].

In this review, the synthesis approach of SNPs, their transportation in the plant, and the roles of SNPs in enhancing plant resistance to abiotic and biotic stress are discussed. It also provides an extensive review of the mechanism of SNPs in improving plant adversity stresses from the aspects of disease, pests, heavy metal, salt, and drought resistance. SNPs participate in enhancing plant resistance to adverse environments. Numerous researchers have studied their resistance modes and mechanisms from different perspectives. However, some mechanisms lack direct experimental evidence. Based on the existing literature, SNPs induce plant resistance to the adverse environments by (1) improving the activities of antioxidant enzymes; (2) increasing anti-stress compounds (e.g., phenolic, flavonols, proline); (3) enhancing photosynthesis; (4) regulating systemic signals. In general, this review describes research insights, existing gaps, and future research directions on SNPs mitigating abiotic and biotic stress on the plant.

## 2. Synthesis and Characteristics of SNPs

**Figure 1:**
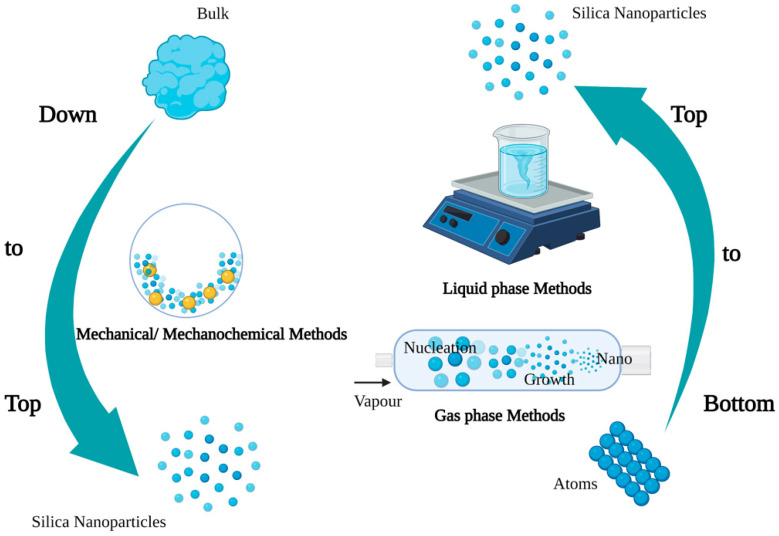
Synthesis methods of nanoparticles (Figure created using BioRender [https://biorender.com/], accessed on 6 February 2022).

SNPs are synthesized via top-down and bottom-up approaches (Figure 1) [26,27]. Top-down strategies of SNPs mainly include mechanical and mechanochemical synthesis methods. The mechanical methods pulverize large solids via ball milling technology, while the mechanochemical approaches assist chemical reactions by mechanical pulverization [28]. The mechanochemical method involves the use of simple equipment at a low cost, and it could produce NPs in batches, but the produced NPs have a wide size range and uneven shape distribution, as well as contain a large amount of impurities [29]. The bottom-up syntheses of SNPs mainly include gas- and liquid-phase synthesis. Gas = phase synthesis has some drawbacks, such as the need for special equipment to produce films and the reaction chamber, and the emission of toxic gaseous by-products [30]. Liquid-phase synthesis covers precipitation, microemulsion, and solution-gel synthesis [31,32,33]. Solution-gel synthesis is one of the most common SNPs synthesis methods. Stöber et al. (1986) reported tetraethylorthosilicate as the silicon source, ammonia as the catalyst, and different alcohol solutions as the solvent to synthesis SNPs with a particle size distribution of 0.05–2 μm [31]. Most mesoporous SNPs are synthesized by a modified Stöber’s method [34]. In addition, the biosynthesis of NPs by microorganisms, algae, and plant compounds is a green and eco-friendly technology [6,35].

SNPs could be classified as porous or nonporous in structure. The synthesis method of SNPs significantly affects their structure with variations in particle size, shape, pore size, and dissolution rate, which influence their application and manner in organisms [34,36]. The size of SNPs is usually governed via adjusting the pH of the reaction solution in the solution-gel method [31]. The particle size and shape of the SNPs greatly impact their cellular uptake and distribution in organisms [37,38]. However, previous studies on the influence of the shape and size of SNPs on organisms have mostly focused on the animal field. In contrast, there are relatively few studies in the plant field. The porosity of Stöber silica could be controlled by adjusting the ratio of water; in conditions of a high ratio of water to TEOS, SNPs with smooth particle surfaces were obtained. In contrast, rough particle surfaces with micropores were synthesized [39]. The type of surfactant added in the liquid phase synthesis process could change the pore size of SNPs [34]. The longer chain lengths of surfactants cause SNPs to have larger pores, and those surfactants with shorter chain lengths cause SNPs to have smaller pores [40]. Pore SNPs are usually used in the delivery of pesticides or herbicides in agriculture. The pores’ size and number of SNPs will affect their degradation rate, which is essential for effective drug delivery [41]. The dissolution rate of SNPs was accelerated as the increase of porosity increased [42]. The dissolution rate of SNPs in plants impacts their effectiveness. Fast-dissolving SNPs could increase plant biomass and fruit yield [11]. Furthermore, in the process of preparing SNPs, some compounds such as (3-aminopropyl) triethoxysilane, oligochitosan, and amino acids could also be added to the synthesis of functionalized SNPs [11,43,44]. For instance, after oligochitosan–nanosilica application, the resistance of chili plants to anthracnose disease rises significantly when compared to single nanosilica treatment [44]. At present, there is a lack of in-depth research about the influence of SNPs synthesis methods on the absorption and distribution of SNPs in plants, plant growth, and stress resistance. In addition, the release behavior of different SNPs (porous, non-porous, surface modification, etc.) in plants needs to be further studied.

## 3. Absorption and Transmission of SNPs in Plants

**Figure 2:**
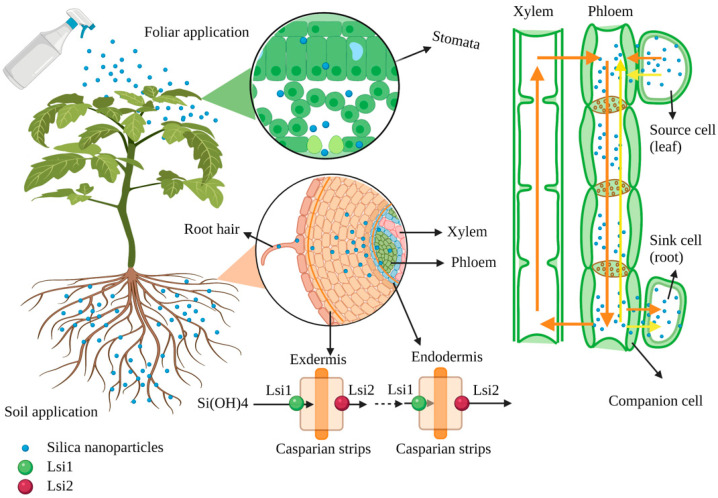
Schematic diagram of the transportation of nanomaterials in the plant (figure created using BioRender [https://biorender.com/], accessed on 6 February 2022).

In agriculture, SNPs can be applied by spraying on the leaves or directly into the root. The SNPs applied by foliar application can enter the leaves and be transported to different parts through the cuticular or stomata (Figure 2). The transport path of solutes via the cuticle has two routes: one is the lipophilic pathway for non-polar solutes via diffusion and penetration, and the other is the hydrophilic pathway for polar solutes via water pores (the effective diameter of these pores is 0.6–0.8 nm) [45,46,47]. In addition, the hydrophilic substances are transported via stomatal pores in the stomatal pathway. However, the unique geometric structure and physiological function of the stomata is complex, and the actual size exclusion limit of the pores penetrated by nanomaterials is still unclear. Hu et al. (2020) investigated SNPs in the guard cells of leaves after spraying SNPs (18 nm) for 3 h [48]. Similarly, SNPs (54 ± 7 nm) could enter the air spaces of the leaf via the stomata and the outer edge of the cell walls [18]. After nanomaterials enter leaves, they could translocate from leaves back to the roots via the phloem [49,50]. In the roots, nanomaterials could form complexes with carrier proteins or root exudates [51]. These complexes can pass through the root cell wall, enter plant cells through intracellular phagocytosis, or directly penetrate the cytoplasmic membrane and then disperse in the cytoplasm [52,53,54,55]. Moreover, nanomaterials can also be transported from the roots to shoots through the xylem [54,56]. Furthermore, the active mode of silicon uptake from roots to shoots via specific transporter proteins includes transport protein (Lsi1), transport protein (Lsi2), and transport protein (Lsi6) [14,57]. SNPs enter the root through the transporter *Lsi1* [54,58]. However, the concentration of SNPs application does not affect the expression of the *Lsi1* gene in plants. Therefore, SNPs enter the plant body mainly through extra mass infiltration [59]. In addition, the expression of silicon transported genes *Lsi1* and *Lsi2* in rice treated with SNPs increases under salt stress [25]. Moreover, *OsLsi1* is a Si-transporting aquaporin (AQP), which was upregulated by Si supplementation, and the AQP could establish the link between Si and molecular signaling [60].

The accumulation and absorption of nanomaterials in plants differ, and the process mainly depends on the type of plant, chemical composition, and the size of nanomaterials [51,61]. Slomberg and Schoenfisch (2012) reported different sizes of SNPs (14, 50, and 200 nm) that could enter *Arabidopsis* roots, but the accumulation of SNPs in root cells decreases with the increase of SNPs size [62]. The efficient transport of nanomaterials into guard cells, extracellular space, and chloroplasts is related to the size and charge of the nanomaterials [48]. Cui et al. (2017) applied different particle sizes (19, 48, and 202 nm) of SNPs to treat rice cell suspension cultures under chromium stress and found that SNPs with small particle sizes had viable rice cells in the suspension [24]. Similarly, the bactericidal effect of SNPs on *Pseudomonas aeruginosa* also increases with the decrease of its particle size [63].

## 4. SNPs Improve Plant Stress Tolerance and Its Mechanism

Plant growth and productivity are limited by extreme environmental conditions, such as pests, disease, heavy metals, salinity, and drought, causing the collapse of entire ecosystems. To overcome biotic and abiotic stresses, plants have evolved various systemic signaling pathways including electricity, calcium, reactive oxygen species (ROS), hydrodynamic waves, and hormones (e.g., jasmonic acid, abscisic acid, and ethylene) [64,65,66]. These systemic signaling changes could be transmitted to the entire plant within a few minutes [67]. Once these system signals are sensed in the plant’s whole-body tissues, the systemic acquired resistance (SAR) is induced, which enables these tissues to withstand stress [68]. SNPs’ application may enhance the plant tolerance to disease, heavy metal, salt, and drought stress by decreased ROS production or hormone levels [18,22,25,69,70].

### 4.1. Disease Stress

SNPs can significantly increase the resistance of crops to different types of diseases, including fungal (*Fusarium oxysporum*, *Aspergillus niger*, *Colletotrichum* sp., *Alternaria solani*), bacterial (*Pseudomona syringae*, *Pectobacterium betavasculorum*, *Dickeya dadantii*), and nematode diseases (*Meloidogyne incognita*) (Table 1). Suriyaprabha et al. (2014) demonstrated that SNPs enhance resistance to *F. oxysporum* and *A. niger* in maize [22]. SNPs induce *Arabidopsis* resistance to *P. syringae* by mediating the salicylic acid signaling pathway to defense resistance [18]. In watermelon, root-dipped treatments with mesoporous SNPs reduced disease severity by 40%, and several stress-associated genes (*CSD1*, *PAO*, *PPO*, *RAN1*) were downregulated in watermelon roots [71].

Pathogens successfully infect the host by penetrating physical barriers, including wax, stratum corneum, and cell walls [79,80]. Si is deposited in the leaf epidermis and stratum corneum, forming a physical barrier to increase the mechanical strength of plants [57,81]. After silicon treatment of rice, the number of penetrative appressorial sites for *P. oryzae* is reduced due to silicon forming a dense silicon layer on the surface of rice that helps prevent or delay the penetration of pathogens [82]. SNPs form a silica layer on the leaf epidermis to increase the resistance to soft rot (*Dickeya dadantii* ) [78]. Rangaraj et al. (2014) reported that corn leaves become harder after SNPs treatment, possibly because of the increase of phenolic substances in leaves, which form an effective physical barrier to protect plants against pests and diseases by promoting the accumulation of silicon [69]. SNPs resist the penetration of parasitic weeds through up-regulated biosynthesis of lignin to enhance the cell wall of the host root [83] On the other hand, silicon might participate in the interaction system between plants and pathogens, activate the host defense response, and induce plants to produce a series of small molecular metabolites to enhance plant disease resistance [22,74]. SNPs significantly increase phenolic content in corn infected by *F. oxysporum*, thus contributing to corn resistance against phytopathogens [22]. In addition, silicon regulates plant resistance enzyme activities [72,75,76]. Recently, El-Shetehy et al. (2020) reported that SNPs activate SAR to decrease the occurrence of diseases caused by *P. syringae* in *Arabidopsis* [18]. SA, ROS, and nitric oxide (NO) are a natural signaling cascade in SAR to control plant pathogens, which are general stress messengers accumulated during pathogens’ attacks [60,84]. SNPs application could alleviate the adverse effect of infection by reducing ROS production and the levels of NO [83]. SNPs reduce disease by inducing plant resistance, but the molecular mechanism of this response still needs to be studied.

### 4.2. Pest Stress

Pests are a vital issue in agriculture because they could damage crops and destroy stored food, leading to the deterioration of food quality and the spread of plant diseases. SNPs have the potential for molecule delivery, release control improvement, and the poisoning of insects [85,86,87]. Though many compounds have a great antifeedant activity to insects, they have some limitations such as short shelf-life, photo ability, lack of availability in large quantities, and application difficulties [88]. However, the use of SNPs-loaded pesticides effectively improves the efficacy of pesticides. The combination of plant extracts and SNPs could enhance their insecticidal activity and shelf life [89]. Similar findings were obtained by Usha et al. (2014), who found that SNPs-loaded α-pinene and linalool increased the antifeedant activity of insects [90]. The trypsin inhibitor applied with SNPs inhibited the growth of *Helicoverpa armigera larvae* [91]. SNPs have a direct toxic effect on *Spodoptera littoralis* (Bosid.) [92], *Sitophilu soryzae* (L.) [93], *Rhizopertha dominica*, *Tribolium castaneum*, and *Oryzaephilus surinamensis* [94]. SNPs cause 90% mortality in *Sitophillus oryzae* [93]. Similarly, Vani and Brindha (2013) found that SNPs cause 100% insect mortality on *Corcyra cephalonica* [95].

The cuticle is the first protection barrier, preventing external compounds from penetrating the insect [96]. SNPs have a large specific surface area, which attaches to insect cuticles, damages the protective wax layer on the stratum corneum, and absorbs the water from the insect body, leading to insect death from dehydration [93,97,98,99,100]. SNPs treatment increases the mortality of the diamondback moth (*Plutella xylostella* L.) to 85% by abrasing the body wall and obstructing stomata [101]. SNPs could also control pests by affecting the life history trait values of peats, including the number of mines, survival rate, and puparia weight [102]. Thus, SNPs have wide potential in the control of pests and are a substitute for traditional pesticides in the future.

### 4.3. Heavy Metal Stress

Heavy metal pollution perturbs the environment, inhibits plant growth and development, and seriously threatens human health. SNPs have an important role in reducing the toxic effects of heavy metals, such as chromium [23], cadmium [103,104,105], mercury [106], lead [107], and aluminum [17] on plants. Chen et al. (2018) found that 5–25 mM SNPs (60–100 nm) decreased the cadmium content of rice by 31.6–64.9% at the flowering stage [108]. SNPs could reduce aluminum toxicity in *Cicer arietinum* plants by promoting seed germination and plant growth [109].

SNPs enhance plant resistance to heavy metals by reducing the accumulation of heavy metals and oxidative stress and activating plant defense systems [17,23,109]. Heavy metal stress induces oxidative stress, leading to the accumulation of ROS, such as superoxide (O^−2^), hydrogen peroxide (H_2_O_2_), and hydroxyl radical (OH^−^), which severely damage the structure, organelles, and functions of plant cells [110,111]. SNPs could activate the antioxidant defense system in roots and leaves, thereby decreasing ROS accumulation in the plant [104,105]. Tripathi et al. (2016) found that SNPs application significantly increases the activity of superoxide dismutase, ascorbate peroxidase, glutathione reductase, and dehydro-ascorbate reductase in maize leaves under arsenic stress [112]. Moreover, glutathione (GSH) is an antioxidant and is crucial for detoxification of heavy metals, which could remove ROS and is a substrate for phytochelatin synthesis [113]. SNPs treatment increases GSH levels to alleviate cadmium toxicity in rice [104]. Under Cd stress, the combination of Si and NO activates the antioxidant defense system of plants by regulating the ascorbic acid–GSH pathway and markers related to oxidative stress in wheat [114]. Tripathi et al. (2016) demonstrated that SNPs application decreases the toxic effects of arsenate on the growth and development of maize by enhancing the components of the ascorbate–glutathione cycle [112]. Furthermore, some specific compounds (e.g., organic acids and phenolic) could chelate metal and scavenge active oxygen under heavy metal stress [115,116]. SNPs enhance maize’s resistance to aluminum toxicity by increasing the accumulation of organic acids (e.g., citrate, lactate, malate, and succinate) and phenols in the roots [17]. Besides, SNPs decrease heavy metal accumulation in plants and restrict the heavy metal stress by promoting photosynthesis, plant growth, and silicon absorbing [117]. Similar results were found in wheat [105]. Moreover, SNPs inhibit the expression of some heavy metal transport and accumulation genes such as *OsLCT1* and *OsNramp5,* which reduce the absorption and accumulation of the heavy metal in the plant [24].

### 4.4. Salt Stress

Salt stress inhibits the growth of plant tissues and parts, and reduces the photosynthetic rate and respiration; leaf growth stops, and plant biomass decreases with the increase of salt concentration and prolonged stress time [118,119]. SNPs could remarkably improve plant resistance to salt stress [120,121,122]. In cucumber, Alsaeedi et al. (2018) found 200 mg·kg^−1^ SNPs (10 nm) increased seed germination rate and the vitality index during salt stress [123]. Similar findings were obtained by Naguib and Abdalla (2019), who found that SNPs application mitigated the adverse effects of salt stress by increasing the activity of antioxidant enzymes [124].

**Figure 3:**
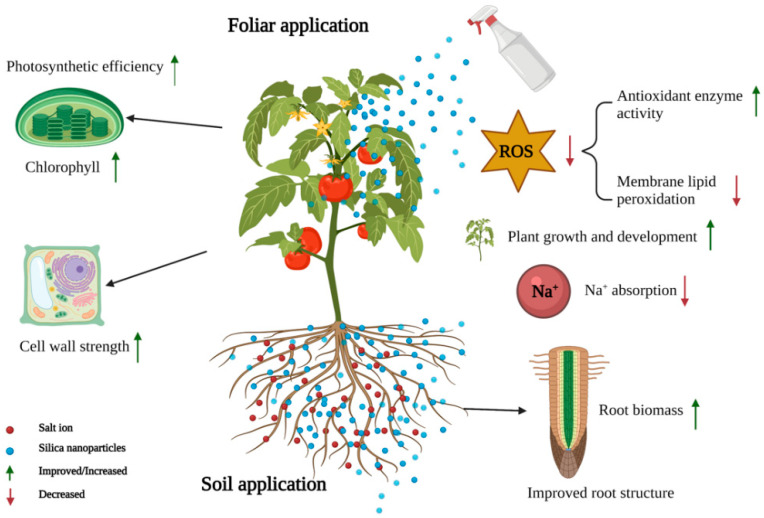
Mechanism diagrams of SNPs alleviating crop salt stress (figure created using BioRender [https://biorender.com/], accessed on 6 February 2022).

The mechanism by which SNPs alleviate plant salt stress is illustrated in Figure 3. The accumulation of plant leaf epidermal wax is related to water-use efficiency and water evaporation [125]. Silicon increases the wax content of the plant epidermis [126]. SNPs application could increase the deposition of irregular crystal wax in the plant cortex under salt stress and maintain the chlorophyll content and relative water content of the leaves, thereby alleviating the effect of salt damage on plants [127]. Plants first respond to salt stress by reducing their leaf surface swelling rate, and then inhibiting photosynthesis. Kalteh et al. (2018) reported that SNPs application increases the chlorophyll content and proline content of basil under salt stress [122]. Moreover, SNPs protect plants from an excess of Na^+^ and Cl^−^ by changing the stomatal conductance of plant leaves, resulting in stable transpiration under salt stress [119]. Plants adjust their cell osmotic pressure to maintain a stable level under salt stress [128]. Proline is an osmotic substance, which usually accumulates under stress conditions and plays an important role in plant osmotic regulation [129]. SNPs increase the content of leaf proline and free amino acids to resist the penetration of NaCl in rice [25]. Furthermore, SNPs supply reduces the influence of salt ions in plants by reducing the absorption of Na^−^ [122]. SNPs change the K^+^/Na^+^ ratio in the cytoplasm by increasing K^+^ absorption and reducing Na^+^, and subsequently reduce the toxicity caused by high concentration of Na^+^. In addition, SNPs activate defense-related enzymes in plants during salt stress to mitigate the damage caused by the accumulation of ROS in plants [25,124]. Moreover, there is a possible involvement of SNPs in the response of plants to stress. Exposure to SNPs resulted in the expression of salt stress genes (*RBOH1*, *APX2*, *MAPK2*, *ERF5*, *MAPK3*, and *DDF2*) being downregulated, enhancing the salt tolerance of tomato plants [130].

### 4.5. Drought Stress

Drought inhibits the germination of plant seeds, impairs plant photosynthesis, and hinders plant growth. SNPs increase the drought tolerance of wheat [131], cherries [132], strawberries [133], and barley [134].

The osmotic adjustment and accumulation of compatible solutes play a role in plant adaptations to dehydration, mainly through the expansion to maintain a specific solute and specific cellular functions [135]. SNPs reduce the osmotic potential of strawberries and barley under drought conditions and improve the ability of tissues to absorb and retain water by adjusting cell osmotic pressure [136,137]. Silicon helps lentil plants absorb and retain more water; this water restoration during drought stress may be related to a double layer of silicon dioxide stratum corneum under leaf epidermal cells [136]. Recent studies on strawberries have also shown that SNPs increase the contents of photosynthetic pigments in strawberry leaves under drought stress, as well as key permeates, such as carbohydrates and proline [133]. Drought stress increases ROS, leading to the over-accumulation of malondialdehyde (MDA), causing plants to suffer oxidative damage [136,138]. SNPs application increases enzymatic antioxidants and decreases the MDA content of plants in the drought stress [70]. Generally, drought significantly reduces leaf expansion, damages photosynthesis, and delays leaf senescence [139]. SNPs reduce the effect of drought on hawthorn by increasing photosynthetic rate and stomatal conductance [140]. SNPs remarkably enhance the SPAD value by 12.54% in wheat under drought stress, thus alleviating the effects of drought stress on plant growth [131].

## 5. SNPs Toxic to the Plant

Currently, the supplementation of SNPs in agriculture has attracted much attention, but the toxicity of SNPs to plants remains controversial. The interaction between plant cells and nanomaterials leads to changes in plant gene expression and related biological pathways, and influences plant growth and development [141]. The SNPs additive E551 is commonly used in food products, which is safe for oral exposure [142,143]. Silica is abundant in human diets and commercial products, and all forms of silica are degraded in the biosphere by hydrolysis of siloxane bonds to nontoxic monosilicic acid, which then re-enters the biosphere [36]. Therefore, SNPs pose a low risk to the environment [144]. However, the potential toxicity of SNPs in plants depends on the conditions of application, as well as their physical and chemical properties [34,41]. The size of SNPs may play a major role in their toxicity due to the large specific surface area and easier penetration into cells [62,145]. In comparison with bulk silica, SNPs (12.5 nm) are more toxic to algae [134]. Van et al. (2008) demonstrated that the ecotoxic effects of SNPs are related to their surface area [145]. On the other hand, the toxic effect of SNPs is likely to be related to the applied concentration, but different plants have variations in degrees of tolerance to SNPs. The 200 mg·L^−1^ SNPs caused cell morphology changes and cytoplasmatic membrane damage in the marine microalgae *Dunaliella tertiolecta* [146]. Slomberg and Schoenfisch (2012) found that at 1000 ppm, SNPs (14, 50, and 200 nm) did not have a toxic effect on *Arabidopsis* growth under hydroponic conditions [62]. Lee et al. (2010) reported that high concentrations of SNPs (more than 2000 mg·L^−1^) may be toxic to *Arabidopsis* root growth [147]. A similar result has been found in sugar beet, in which 2 mM SNPs increased the MDA contents under drought stress [70]. The rapid generation of ROS is a common plant response to stress conditions. Plants would produce some stress signaling including antioxidant enzymes and metabolites, to scavenge ROS under various types of stresses. However, SNPs may perturb the biological pathways of plants. In maize leaves, amino acid metabolism, methane metabolism, carbon metabolism, and increased defense responses occur after root exposure to SNPs [148]. Currently, few studies have documented the relationship between plant health and the size, dosage, and accumulation of SNPs in plants. Thus, the toxicity of SNPs to plants needs further study.

## 6. Conclusions

SNPs play an important role in alleviating the adverse effects of environmental stress on plants [18,19,107,132]. Based on relevant studies, we proposed some potential mechanisms that may explain how SNPs increase plant resistance to biotic and abiotic stresses. First, SNPs could promote plant growth and development by increasing the photosynthesis and nutrient absorption rate of plants in adverse environments. Second, SNPs could form silica precipitates on the surface of plants and increase the mechanical strength of plants that build the first protective barrier for plants against environmental stressors. Third, SNPs could induce plants to produce a series of defensive compounds, which change the internal environment of the plant that directly defends against stresses. Finally, SNPs enhance plant tolerance to adverse stresses by stimulating the antioxidant systems of the plant to reduce the accumulation of ROS under stress and induce the expression of defense genes.

However, the difference in SNPs chemistry, size, shape, and electromagnetic properties resulted in differences in findings on the effects and mechanisms of SNPs in alleviating plant stress. Thus, the following problems should be the focus of future research on SNPs to improve plant resistance during stress conditions:

1. The study of the behavior of SNPs in plants and their interaction mechanism, influence, and agricultural application is at its early stage [21,93]. Previous studies have mainly focused on enhancing physical barriers, promoting plant growth, inducing plant resistance, and activating resistance enzyme activity, but rarely involved the effect of SNPs on plant metabolites and soil microbial community under stress. The “SNPs–plant–soil–microorganism” system should be considered as a whole system, and in-depth research should be conducted on the molecular mechanisms of SNPs that increase the plant’s resistance to adversity from the physiological, biochemical, molecular, proteomic, and metabonomic levels through omics methods.
2. The combination of SNPs and other methods such as surface modification of SNPs, SNPs loaded with fungicides, and the combination of SNPs with beneficial microorganisms could increase the effect of SNPs in improving plant stress.
3. It is necessary to establish a synthesis–structure–toxicity relationship. The synthesis of SNPs affects their structure and surface chemical characteristics, thereby controlling the degradation of SNPs in plants. After being absorbed by plants, SNPs exist internally within plants’ and are slowly released, thus influencing the life activities of plants. The release of SNPs and their biological toxicity need further attention. The interaction between SNPs and environmental microorganisms also requires special attention.

## Figures and Tables

**Table 1:** Application of SNPs in controlling plant diseases.

Size and Concentration	Plants	Applied Method	Disease	Mechanism	References
20–40 nm, 5, 10, and 15 kg·ha^−1^	Corn	Soil application	*Fusarium oxysporum*, *Aspergillusniger*	Increase phenolic content, activated defense-related enzymes	[22]
30–50 nm 60 mg·L^−1^	Chili	Foliar spray	*Colletotrichum* sp.	_	[44]
100, 200, 300, and 400 mg·L^−1^	Tomato	Foliar spray	*Alternaria solani*	Killed germs	[72]
<50 nm 50, 100, 150, and 200 µL·L^−1^	Potato	Soaked potato tubers	*Rhizoctonia solani*	activated defense-related enzymes	[73]
36–39 nm 500 mg·L^−1^	Watermelon	Root dip	*Fusarium oxysporum f.* sp. *niveum*	Reduced the expression of stress-related genes	[71]
30–60 nm, 1.5 and 3 mM	Broad bean	Foliar spray	*Botrytis fabae*	Increased defense compounds and activated defense-related enzymes	[74]
54 ± 7 nm, 25, 100, 400, and 1600 mg SiO_2_·L^−1^	Arabidopsis	Foliar spray	*P. syringae*	Induce plant resistance	[18]
5–15 nm, 100 and 200 mg·L^−1^	Beet	Foliar spray and Seed soaking	*Meloidogyne incognita*, *Pectobacterium betavasculorum*, and *Rhizoctoniasolani*	Promoted growth, improved photosynthesis, activated defense-related enzymes	[75]
15 nm 50 mg·L^−1^	Rice	Foliar spray	*Fusarium fujikuroi*	Improved peroxidase activity	[76]
1500 mg·L^−1^	Watermelon	leaf immersion method	*Fusarium oxysporum*	_	[11]
20, 40.2, 70.2, and 95.5 nm 0.5, 2.5, 5, and 10 ppm	Maize	In vitro experiment	*Harpophora maydis*	inhibited the mycelia growth	[77]
0, 7.5, 15, 22.5, and 30 ppm	Phalaenopsis	Foliar spray	*Dickeya dadantii*	Promoted growth	[78]

## Data Availability

Not applicable.
